# Understanding Myeloma-Related Information Needs and Communication Preferences Within Black American Communities: An Exploratory Study

**DOI:** 10.1007/s13187-024-02480-3

**Published:** 2024-08-16

**Authors:** N. S. Esquivel, J. P. Tzeng, K. Treiman, C. H. Husick, J. Sheridan, L. Ortiz-Ravick, M. Sae-Hau, L. Brown, K. DeMairo, N. Bell, K. Disare, E. S. Weiss

**Affiliations:** 1 RTI International, Research Triangle Park, Durham, NC USA; 2 The Leukemia & Lymphoma Society, Rye Brook, NY USA

**Keywords:** Myeloma, Black American, Communication preferences, Information needs

## Abstract

Myeloma is the third most common blood cancer and one of the most complex and expensive cancers to treat. Black Americans face health disparities related to myeloma incidence, age at diagnosis, access to novel treatments, and mortality. To help reduce health disparities among Black Americans through education and outreach, the Leukemia & Lymphoma Society has implemented its Myeloma Link initiative. In 2022, a formative, qualitative evaluation was conducted across the 15 U.S. cities that implemented Myeloma Link to better understand the information and communication needs and preferences of three groups: patients, community members, and primary care providers (PCPs). Data collection included interviews with eight patients, two focus groups with a total of ten community members, and interviews with six PCPs. Patients expressed wanting information about treatment experiences, including clinical trials, and emotional and peer support services, particularly from other Black American patients. Community members were largely unfamiliar with myeloma and desired outreach via trusted community organizations about disease signs and symptoms. Both groups discussed the importance of self-advocacy within the current healthcare system and wanted actionable messaging, rather than messaging leading with disparities statistics. PCPs described systemic capacity and time challenges in the context of needing to address more frequently encountered health conditions; nonetheless, PCPs welcomed information and brief trainings about myeloma diagnosis and treatment options, referrals to specialists, and how to improve care, prognosis, and caregiver support. Findings underscore the importance of outreach initiatives such as Myeloma Link to help meet these needs and reduce health disparities.

## Introduction

Myeloma is the third most common blood cancer in the USA, with over 35,000 new cases and 12,590 deaths in the USA in 2023 [1]. It is one of the most complex and expensive cancers to treat [2]. Recent advances in treatment, including immunomodulatory drugs and chimeric antigen receptor T-cell (CAR-T) immunotherapy, have substantially improved survival [3, 4]. However, racial disparities persist in myeloma diagnosis, treatment, and survival, as not all have benefited equally from these advances [5–10].

Compared with patients who are White, patients who are Black American (BA) have a two-fold higher incidence of myeloma (15.9 vs. 7.5 cases per 100,000), higher mortality rate (5.6 vs. 2.4 per 100,000) [11], are diagnosed at younger ages [12], and experience a longer time from diagnosis to novel therapy initiation [5] and to hematopoietic cell transplant [13]. When BA patients receive equal and high-quality care, clinical outcomes can be similar or even better than those of White patients [5, 10, 14, 15]. The sources of racial disparities in myeloma are multifactorial and include an interplay of health care factors (e.g., access, utilization) [5, 6], socio-economic status [7], and differences in disease biology (e.g., genomic and molecular tumor differences) [5, 6, 10, 13, 16, 17].

Current literature underscores the need for information, education, and communication to increase awareness of myeloma symptoms and treatment options in both the BA community and among health professionals who care for this community, to ensure timely and equitable access to therapies. While the National Cancer Institute, the Centers for Disease Control and Prevention, and other organizations address cancer disparities broadly, to our knowledge, few initiatives exist that specifically address racial disparities in myeloma in the USA. Here, we describe the Leukemia & Lymphoma Society’s (LLS) Myeloma Link initiative and present formative evaluation findings on the information and resource needs, communication preferences, and perspectives of BA patients and community members, as well as primary care providers (PCPs). We discuss how these findings can be applied to improve outreach, education, and communication interventions to address myeloma disparities.

### Myeloma Link

LLS established Myeloma Link with a goal of reducing myeloma-related health disparities experienced among BA patients and communities. Specifically, the initiative aimed to: (1) increase awareness of myeloma and other blood cancers in communities that have a high proportion of BA residents; (2) connect BA blood cancer patients and their caregivers with LLS educational and supportive services; and (3) build relationships with community-based organizations and PCPs to raise awareness of myeloma and of LLS educational and support services. Myeloma Link was piloted in 2017 and has since expanded to 15 cities, with online and social media programming that reaches across the nation. From July 2022 to June 2023, LLS implemented over 350 activities focusing on community outreach, patient education, and healthcare provider outreach and education to over 75,000 individuals. In addition to providing patient and healthcare provider educational programming, LLS often incorporated myeloma-focused education into popular activities and events. Activities have included: sharing information through churches (“Myeloma Sundays” and “Health Sermons”); programs in other familiar settings to raise awareness among the lay community and to educate patients; “lunch and learns” for PCPs; and outreach conducted via community volunteers and in collaboration with trusted institutions within BA communities (e.g., Meals on Wheels, National Coalition of 100 Black Women, nurses’ associations, libraries, local fraternities and sororities, faith-based groups, senior centers, barbershops).

In 2022, LLS contracted with RTI International to conduct a formative evaluation of Myeloma Link and identify opportunities to strengthen program reach, delivery, and impact. At the time of the evaluation, Myeloma Link was being implemented within 15 US cities. The evaluation sought to explore the following questions: (1) What are the information and communication needs of BA patients and community members about myeloma and how do they want to receive myeloma information and education? (2) What are PCPs’ needs related to diagnosing patients with myeloma?

## Methods

We conducted a qualitative, exploratory study to understand the information needs and communication preferences of BA patients, BA community members, and PCPs. We utilized a professional recruitment firm to identify a sample of adult community members and myeloma patients (ages 21 years and older) who were BA, English-speaking, and living in cities where Myeloma Link was implemented; we also used convenience sampling to recruit one male patient to supplement the female participants that the recruitment firm found for the initial wave of patient interviews. Patients were in their 40 s to 70 s and their education levels spanned from some college/technical degree to post graduate degrees. Community members were in their 20 s to 70 s, and their education levels spanned from some college/technical degree to post graduate degrees. Additionally, we recruited a convenience sample of PCPs who were practicing in those communities at the time of our data collection through our team’s professional networks. From July to November 2022, we conducted 2 waves of in-depth interviews (virtually) with patients (*n* = 8 across both waves) and 2 focus groups with community members (virtually) (*n* = 10) from 9 Myeloma Link cities, and interviews (virtually) with PCPs (*n* = 6) from 2 Myeloma Link cities.

Patient interviews explored experiences of being diagnosed with myeloma, barriers to care, sources of health information, unmet needs for information and support (addressed in wave 1 interviews), communication preferences for learning about treatment and support, and what would motivate patients to connect with LLS (addressed in wave 2 interviews) (Table 1). Focus groups with community members explored awareness of myeloma, access to healthcare, and preferences for learning about myeloma. In interviews with PCPs, we asked about their awareness of the signs and symptoms of myeloma and preferences for learning about myeloma.

**Table 1:** Overview of data collection by participant type and sample interview/focus group questions

Audience type	Mode of data collection	*N*	Geographic areas represented (# and sex of participants)	Sample interview/focus group questions
Patients with myeloma who are Black American	Virtual individual interviews	8 (2 waves; 2 individuals participated in both waves)	Wave 1: Atlanta *(1 F)* Dallas *(1 F)* Houston *(2 F)* Raleigh-Durham *(1 M)* Wave 2: Atlanta *(1 F)* Chicago *(1 M)* Oakland *(1 M)* Raleigh-Durham* (2 M)*	• What challenges did you face when trying to see a doctor about your symptoms? • What kind of resources or information about myeloma do you wish you had received after your diagnosis? • Where do you go to get trusted information about your health? • How would you like to hear more about a program such as Myeloma Link? What would be the best ways to draw your attention? • What kinds of message or information about a program like Myeloma Link would be most compelling to you and make you interested in learning more about the resources, services, and support offered by LLS? • What do you think about using a celebrity or a non-celebrity patient/caregiver as the spokesperson for a Myeloma Link campaign?
Black American residents in a city with Myeloma Link	Virtual focus groups	10 (2 groups)	Group 1: Houston *(1 F)* Bronx *(1 F)* Chicago *(2 F)* St. Louis *(1 F)* Group 2: Atlanta *(2 F)* Baltimore *(1 F)* Dallas *(2 F)*	• How many in the group have heard of a cancer called myeloma? • What are your thoughts about barriers to care that contribute towards health disparities among myeloma patients who are Black American? Have you experienced or witnessed barriers to healthcare in your community? • What about myeloma? Do you think your family, friends, and neighbors need to know? • How would you frame this information in a way that would resonate with your family members, friends, and neighbors? • How would you present this information so that people in your community pay attention and remember the main points?
Primary Care Providers (who serve BA patients)	Virtual individual interviews	6	Chicago *(2 F)* Raleigh-Durham *(4 F)*	• How familiar would you say you and your colleagues are regarding the signs and symptoms of myeloma? • What are effective ways to share information about myeloma and organizations like LLS with other Primary Care Providers like yourself?

^Note.*F denotes *female, *M *denotes male^

Provider interviews lasted 30 min, patient interviews lasted 60 min, and focus groups lasted 75–90 min. Participants provided verbal consent to participate before each interview and focus group. The interview and focus group discussions were recorded with participant permission and transcribed. Participants received a $150 incentive. This study was deemed not to be human subject research by the RTI International Institutional Review Board because it was considered a program evaluation based on the US Code of Federal Regulations (45 CFR 46.102) (IRB ID: STUDY00021924).

We used confirmability methods (i.e., peer debriefing) [18] to ensure the validity of the qualitative data: The lead interviewer and notetaker would meet after each interview to discuss and reach consensus on salient take-aways. We conducted rapid qualitative analysis using inductive-deductive coding to identify trends and consistencies in participants’ responses using a matrix approach [19]. We reviewed each transcript and coded responses in categories that aligned with our core research questions. We synthesized data within each code to identify the information and support needs and communication preferences of patients, community members, and healthcare providers.

## Findings

We present the most salient themes related to the information and resource needs and communication preferences of patients, community members, and PCPs.

### Patients’ Information and Resource Needs

#### Interest in hearing from and connecting with BA patients

Patients expressed an interest in learning from peers about treatment options, current myeloma research, and clinical trials. They wanted to hear patient testimonials regarding experiences with treatment (e.g., participating in clinical trials, receiving a stem cell transplant, “watchful waiting”), particularly from other BA myeloma patients. Hearing from patients with whom they identified increased trust and was important given the historical context of discrimination in the healthcare system, especially when thinking about clinical trial participation. Patients also expressed interest in learning about the financial supports available from LLS, due to the sudden and lengthy financial burden of treatment.

*You may get more of a sense of truth coming from someone you know, or your same race… If it’s [a testimonial] coming from someone of the same race and you know the (side) effects…it may be similar [treatment effects], more so than that of someone [who] is not of the same race. – Patient*

#### Need for Social and Emotional Support

Patients described the importance of self-advocacy and emotional support during their cancer care. For example, one patient described having to push for a second opinion and further diagnostic testing with a specialist after his symptoms were dismissed by the PCP. When available, patient navigators provided emotional support and increased patients’ knowledge about their diagnosis, treatment options, and medication side effects. Patients stressed the need for emotional support, particularly through racially diverse support groups and peer-to-peer support, and some patients specifically wanted opportunities for in-person support. Patients desired social connectivity to combat loneliness when going through treatment. Finally, the Black church emerged as providing an important, positive space for patients to rely on their belief system and trusted community while making decisions about and undergoing treatment.

*My mother – she was the only family member who knew the struggle I was going through…I would have liked for more family members to know for additional prayers. - Patient*

### Patients’ Communication Preferences

#### Importance of Engaging Culuturally Relevant Institutions

Participants emphasized the importance of collaborating with Black-owned and patronized businesses and organizations (e.g., churches, community health clinics) to increase visibility of Myeloma Link and LLS within their communities. Several participants emphasized the importance of the Black church as a place to share health information.

*[Learning about Myeloma Link at a local church] would definitely be something that will be positive, because even if the person isn’t dealing with it themselves personally, they may have a family member … that’s dealing with it and they want to get information” – Patient*

#### Effective Message Framing and Delivery

Patients preferred not to hear messages that lead with statistics highlighting disparities, which sounded *“like a victim statement”* and did not help them understand and navigate their myeloma diagnosis. They preferred to hear from a relatable patient or caregiver with a specific call-to-action. Patients wanted messaging about myeloma to resonate with patients’ experiences with diagnosis or treatment, or from a campaign spokesperson who shares common experiences (e.g., a patient with myeloma or a caregiver who is BA). A celebrity spokesperson could potentially be effective, if the celebrity is a myeloma patient or caregiver. There was a clear preference for spokespersons who have a direct connection to myeloma, regardless of race.

*It is more important for a spokesperson to have had myeloma than be the same race as me…[myeloma patients who are BA] need to know how to get treated and what questions to ask. Where to go for reputable information. - Patient*

### Community Members’ Information and Resource Needs

#### More Awareness About Myeloma and Available Resources

The focus groups validated the need for greater awareness about myeloma among BA community members, as about half of the participants had not heard of myeloma. Those who had heard of myeloma typically had a loved one with a diagnosis. Focus group participants expressed the importance of raising awareness, including of signs and symptoms, what treatment involves, and available financial, support and informational resources.

*I think it’s important to be informed so you can advocate for yourself and for your loved ones. - Community Member*

#### Repercussions of PCPs’ Limited Bandwidth

Focus group participants also expressed broader concerns about healthcare and wanted providers to take their health concerns more seriously. They shared experiences of having their health concerns brushed aside or not fully addressed. For example, some participants described how some providers “push patients off” by not scheduling timely follow-up visits about a health concern. Others acknowledged constraints faced by their providers, including high patient volume and limited time for patient visits, which they understand can be a barrier to thorough discussion and examination.

*I don’t know if they’re overworked but like you tell them something and they push you off to the side and just say, ’well just come back and we’ll look at it in a few months’ and sometimes it’s too late for people.- Community Member*

### Community Members’ Communication Preferences

#### Trusted Sources in Community as Messengers

Community members expressed that health-related information is most effective when coming from trustworthy sources, such as someone relatable who also has relevant experience (e.g., a trusted health care provider, a myeloma patient from the community). They shared that myeloma materials should reflect BA individuals, in ways that are compelling and clearly demonstrate information relevant to their community. Participants discussed ways to communicate with different age groups and the importance of identifying the best channels to reach the specific priority audiences (e.g., social media for younger age groups). Participants said that gathering places that “really get into the heart” of where the BA community feels safe may resonate with all age groups and especially older adults. Suggestions included recreation centers, assisted living homes, soup kitchens, neighborhood associations, barber shops, and other gathering places, such as the community gym and the YMCA.

*I think awareness through these social media stars will have [young] people pay attention…more than … in a doctor’s office… Also, ‘in the street’ type of awareness, when you see people at tables and when you speak to the people face to face. – Community Member*

#### Preference for Call to Action Over Statistics

Participants shared that information about the disproportionate impact of myeloma among BAs did not capture their attention and can be off-putting, as they hear similar statistics about health disparities for many other health conditions, including diabetes, high blood pressure, and other cancers. Instead, they preferred to hear messages that provide information regarding what they can do if they experience potential signs and symptom of myeloma (e.g., call the doctor, request specific blood or imaging tests) or are diagnosed with myeloma (e.g., contact LLS).

*I’m really not surprised at the statistics… a lot of times we don’t have the access to healthcare that others do, so we end up…maybe waiting until the last minute to get help…[We need to be] creating awareness, creating conversations between family, friends and the community. – Community Member*

### Primary Care Providers’ Information and Resource Needs

#### Additional Resources About Myeloma for Providers

Most PCPs said that they are familiar with myeloma but do not encounter it often in primary care. They expressed a need for information, resources, and brief trainings about myeloma diagnosis, treatment options, referrals to specialists, and how to improve patient care and caregiver support. They suggested provider resources including a provider “cheat sheet” about myeloma diagnosis and clinical pathways and resources they can share with patients and caregivers (e.g., information about support groups and organizations offering patient support).

*I feel like in general, [we need] those services where you can reach out to a specialist and just have a…conversation, and it helps figure out how much you need to prioritize advocating for [your patient] to be seen quickly… – PCP*

#### Systemic Barriers to Timely Diagnosis

PCPs described systemic challenges in the delivery of healthcare that can impede timely diagnosis of myeloma, such as high patient caseloads, limited time to evaluate possible myeloma signs and symptoms, limited number of in-network specialists, staffing issues (e.g., turnover and shortages), and increasing administrative burdens. PCPs must address many other health conditions and cannot prioritize identifying potential myeloma (or blood cancers in general).

*There are really big systemic problems that need to be solved before we can get clinicians to change. They’re not lazy; they’re overworked. Most people will just do the easy thing and prescribe opioids – labelling them as chronic pain patients. - PCP*

## Discussion

BA communities need and want effective information, education, and communication strategies to increase awareness of myeloma and facilitate timely diagnosis and entry into care. Myeloma Link is one such program implemented by LLS to reach BA communities and myeloma patients. Across patients, community members, and PCPs, we found genuine interest in learning more about myeloma and identified potential strategies to improve the content and delivery of information, education, and messaging based on the needs and preferences of each stakeholder group.

Patients were eager for more information about their diagnosis and treatment options, as well as current myeloma research and clinical trial opportunities. Patients especially valued hearing from other BA patients about their experiences with myeloma treatment. Patients also stressed the importance of emotional support as the patient experience can be isolating. They valued opportunities for patient navigation and peer support, particularly groups that are in-person and racially representative. These findings are consistent with prior research about unmet information needs among cancer patients [20] and the critical role of supportive care [21].

Among community members, we found limited awareness of myeloma but little surprise about the disproportionate impact on BA communities, as they are accustomed to hearing about health disparities. Patient and community outreach and education should feature action-oriented information rather than focusing on disparities, which community members may tune out. Participants stressed the importance of receiving myeloma information from trusted sources in the community. This is consistent with prior research on strategies for delivering cancer information to BA communities focused on prostate [22, 23] and colorectal cancer [24], through trusted sources including personal physicians, clergy, family (especially spouses) and cancer survivors [22, 23], and messages culturally tailored and that support patient empowerment [23, 24].

Our study also included PCPs, who are typically the first point of contact for patients experiencing potential myeloma signs and symptoms that require evaluation and referral to specialists. For patients with myeloma, delayed diagnosis poses a challenge to improving care and outcomes, as patients and healthcare providers often do not recognize myeloma symptoms [25, 26]. PCPs stated that they do not routinely encounter patients with myeloma and would benefit from educational opportunities and resources both for themselves and to share with patients and family members.

This was a small-scale qualitative study, and participants did not represent all the diverse perspectives of their communities. Importantly, all community member focus group participants were female, and males are likely to have different perspectives. However, we reached saturation among the focus group participants as similar themes arose across both groups. Finding myeloma patients who are BA and were willing to participate in interviews was another challenge. Despite the sampling limitations, the study provides insights that can guide information, education, and communication efforts to address myeloma disparities. Further research is warranted with larger sample sizes to confirm and refine our formative evaluation findings.

## Conclusion

Our findings underscore the importance of initiatives such as Myeloma Link to increase awareness of myeloma and timely access to quality myeloma care within BA communities. Findings highlight that collaborating with trusted community-based organizations and integrating myeloma education into established community events is a promising approach to raise community awareness. Culturally relevant resources about treatment, clinical trials, and social support are needed. An approach featuring BA voices and focusing on action(s) to be taken is likely to capture attention and effectively convey information to ensure that communication messages and materials are culturally relevant and resonate with the audience. PCPs can play an important role in ensuring timely diagnosis and treatment. However, providers in the primary care setting have limited experience with myeloma and must address many other priorities within significant time constraints. Consequently, educational resources for PCPs must be brief and easily accessible (e.g., available in different modes and on-demand).

## Data Availability

Qualitative data from this study cannot be shared because of confidentiality considerations.
